# Hemolytic Anemia in the Setting of Atypical Pneumonia: A Case of Cold Agglutinin Disease

**DOI:** 10.7759/cureus.39734

**Published:** 2023-05-30

**Authors:** Abdelwahab Jalal Eldin, Roshni Thomas, Gary Gibson, Davis Abongwa, Israa Hassan, Mpey K Tabot Tabot, Gagan Singh, Ravi Sarma

**Affiliations:** 1 Internal Medicine, Howard University Hospital, Washington, D.C., USA; 2 Hematology and Medical Oncology, Howard University Hospital, Washington, D.C., USA

**Keywords:** severe anemia, cold agglutinin syndrome, cold agglutinin, immune hemolytic anemia, autoimmune hemolytic anaemia

## Abstract

Cold agglutinin hemolytic anemia (cAHA) is a rare autoimmune disorder characterized by the production of cold agglutinins. We present a case of secondary cAHA in a 23-year-old female with severe anemia and unexplained hemolysis. The patient exhibited findings indicative of hemolysis and a positive direct antiglobulin test (DAT) with complement alone. Additional investigations revealed incidental lung infiltrates, negative serology for infections and autoimmune diseases, and a low cold agglutinin titer. The patient showed a favorable response to doxycycline and supportive therapy, including multiple packed red blood cell transfusions. At the two-week follow-up, the patient had a stable hemoglobin level with no evidence of ongoing hemolysis. This case highlights the importance of considering secondary cAHA in patients with cold symptoms or unexplained hemolysis. Primary cAHA patients may require more aggressive treatment, including rituximab and sutilumab.

## Introduction

Cold autoimmune hemolytic anemia (cAIHA) is a rare hematologic disorder with an estimated incidence of 0.45 to 1.9 cases per 1 million persons per year. The disease destroys red blood cells through autoantibodies at low temperatures. The two types are primary cold agglutinin disease (CAD) and secondary cold agglutinin syndrome (CAS), with the latter caused by underlying infections, autoimmune diseases, or malignancies [1]. Due to the potential for complications such as severe anemia and thrombosis, timely diagnosis and treatment are crucial [2]. This case report presents a challenging case of a young female with severe cAIHA, highlighting the diagnostic and therapeutic considerations for this rare and complex condition.

## Case presentation

A 23-year-old female presented to the emergency room with fatigue, body ache, and lightheadedness for 10 days. She reported a recent history of upper respiratory tract infection, which was treated with cefdinir, and a remote family history of mycoplasma pneumonia. On examination, she appeared pale and jaundiced. Her vital signs were notable for a blood pressure of 105/55 mmHg, heart rate of 101 beats per minute, respiration rate of 27 breath/minute, and oxygen saturation of 96% on 3L nasal cannula.

Lab studies revealed leukocytosis of 32×10^9^/L with absolute neutrophilia, profound anemia with a hemoglobin (Hb) of 2.9 g/dL, and hematocrit of 8.8% compared to a Hb of 10.9 g/dL five months prior. The anemia workup showed high levels of serum iron, ferritin, and adequate transferrin, as well as elevated total bilirubin with normal direct bilirubin (Table 1).

**Table 1:** Laboratory investigations

Investigation	Results	Reference
Absolute neutrophil	23.9	1.3-7 × 10^9^/L
Procalcitonin	.57	< .5 ng/mL
MCV	77	77-94 fl
Serum iron	345	25-160 ug/dL
Ferritin	3064	20-400 ng/dL
Transferrin	238	180-362 mg/dL
Total bilirubin	5.7	.2-1.2 mg/dL
Direct bilirubin	1.1	0-.2 mg/dL
Reticulocyte	3.5%	.5-2%
LDH	1073	100-250 IU/L
Haptoglobin	<30	36-195 mg/dL
Serum B12	546	200-950 pg/mL
ANA	Negative	
ANCA	Negative	
EBV IgM	Negative	
Parvovirus IgM	Negative	
HbsAg	Negative	
Urine pregnancy	Negative	
MCV: mean corpuscular volume; LDH: lactate dehydrogenase; ANA: Antinuclear antibody; ANCA: Antinuclear cytoplasmic antibody; EBV: Epstein–Barr virus; HbsAg: Hepatitis B surface antigen		

**Figure 1:**
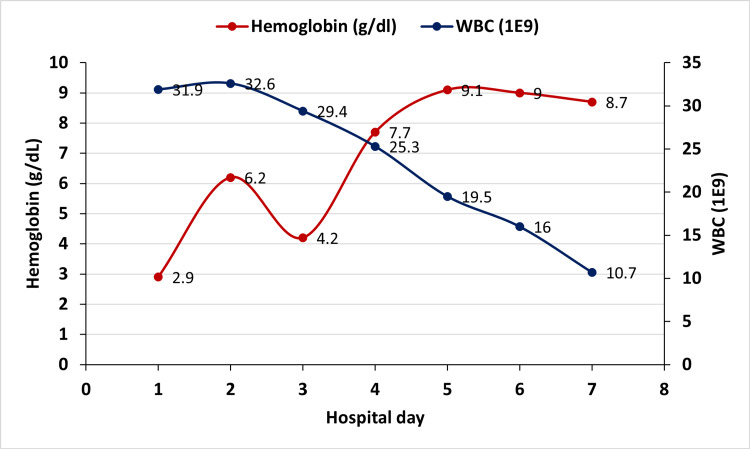
Trends in Hemoglobin and White Blood Cell Count during Hospitalization

The patient received 2 units of packed red blood cells, which resulted in an initial increase in hemoglobin to 6.2 g/dL followed by a drop to 4.2 g/dL in 24 hours (Figure 1).

A hemolysis workup showed reticulocytosis, high lactate dehydrogenase, low haptoglobin, and positive direct antiglobulin tests for anti-C3 and polyspecific antibodies, but was negative for anti-IgG antibodies. A peripheral smear showed microcytic, normochromic RBCs with red cell agglutination and rouleaux formation, leukocytosis with neutrophil predominance and atypical lymphocytes suggestive of reactive B cells, while platelet count was adequate. The patient received a total of 6 units of warm-packed red blood cells for symptomatic anemia to maintain a hemoglobin level above 7 g/dL.

**Figure 2:**
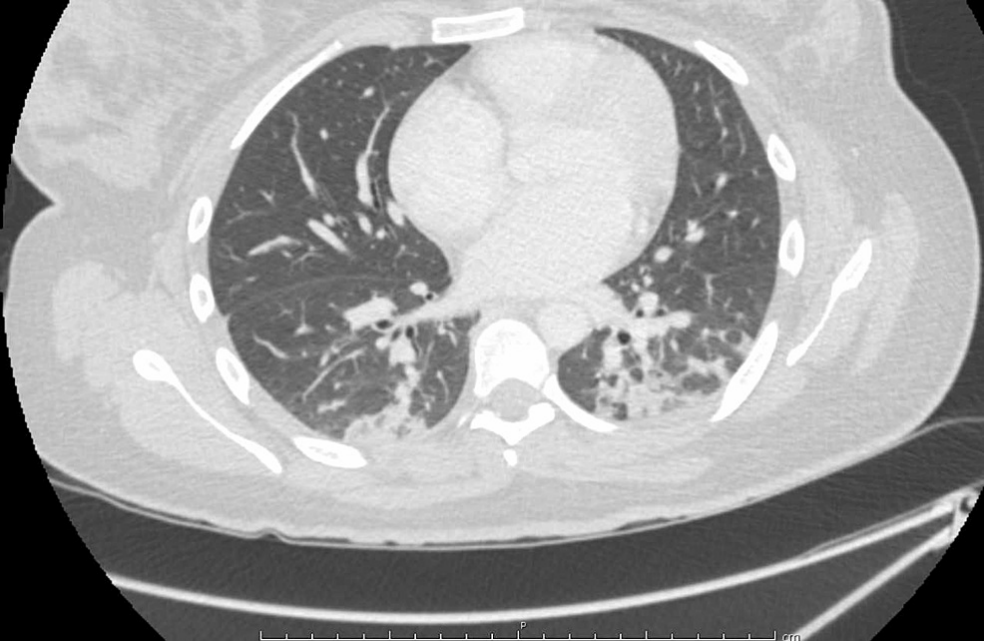
Bilateral airspace disease

On day 2 of admission, she complained of chest discomfort, and elevated troponin levels were detected on laboratory studies. However, an echocardiogram and chest X-ray were unremarkable. The patient was then evaluated by our cardiac team, who recommended correcting the anemia. The patient underwent a workup for secondary cold agglutinin syndrome (CAS). CT with contrast of the chest/abdomen/pelvis showed bilateral lung airspace disease and trace bilateral pleural effusion (Figure 2).

Tests for mycoplasma IgM and legionella urine antigen were negative, as were the blood cultures and autoimmune workup. The patient was treated with oral doxycycline and discharged on day 6 with a hemoglobin level of 8.7 g/dL. Follow-up at 2 weeks showed no evidence of hemolysis, and her hemoglobin level was 10.7 g/dL.

## Discussion

Cold agglutinin autoimmune hemolytic anemia (cAIHA) should be suspected in patients presenting with cold symptoms or unexplained hemolysis, as was the case with our patient. The diagnosis of cAIHA is primarily based on the presence of hemolysis, a positive Coombs test, and a cold agglutinin titer of >63 at 4°C [1,3]. Our patient presented with overt hemolysis with a significant drop in hemoglobin that required multiple warmed blood transfusions, RBCs agglutination on peripheral smear, positive DAT polyspecific and anti-C3 tests, negative DAT anti-IgG, and a low cold titer. Although an incidental lung infiltrate was noted on CT, it was negative for malignancy. The patient’s mycoplasma IgM was negative twice, and tests for EBV IgM, COVID-19 PCR, HIV serology, ANA, and ANCA were also negative. She had received her COVID vaccination booster 3 months prior to the presentation.

There is a possibility that the cold titer test was not performed properly, as it is critical to keep the blood sample at 37-38°C from sampling to preparation of the serum to avoid false low results [1,3-6]. Another possibility is that her late presentation during the recovery phase, as indicated by her low hemoglobin on admission, resulted in a low titer.

Our patient’s presentation and workup suggest secondary CAS precipitated by atypical pneumonia. Secondary CAS due to infections are typically polyclonal and transient, resolving with the resolution of the infection, spontaneously or with antibiotics if indicated [7]. Kanagavelu et al reported a case of mycoplasma-induced CAS that resolved completely after treatment of the underlying infection [2]. Our patient responded well to doxycycline and multiple pRBC transfusions, and her 2-week follow-up after discharge showed stable Hb and no evidence of hemolysis.

Other reports have documented infection-induced CAS caused by EBV, adenovirus, and HIV [1,8]. Our patient had negative EBV and HIV results, and further infectious workup was not conducted as she was discharged early while some of the initial viral workup was pending.

In contrast, most cases of primary CAD are low-grade lymphoproliferative disorders that lead to the production of cold agglutinins (the majority of which are monoclonal IgM) directed against the ubiquitous "I" antigens on the surface of RBCs in the body extremities at low temperature, depending on thermal amplitude. This leads to complement activation and opsonized RBCs are phagocytosed mainly in the liver (extravascular hemolysis) [1,4].

The management of cAIHA involves avoiding cold exposure until recovery and providing warm blood transfusions in symptomatic patients. Symptomatic primary CAD patients may require temporary measures including plasmapheresis, but the cornerstone of management targets pathogenic B cells with rituximab-containing therapy. Recently, Sutimlimab, a complement inhibitor, has been approved for use in CAD as a secondary measure, temporarily halting hemolysis and decreasing the need for RBC transfusion [3,8-10].

## Conclusions

In conclusion, cAIHA is a rare condition that can present with severe hemolytic anemia and should be considered in patients with cold symptoms or unexplained hemolysis. The diagnosis is based on evidence of hemolysis, a positive Coombs test, and a cold agglutinin titer of >63 at 4°C. In primary symptomatic CAS, rituximab is the cornerstone of management, while in secondary CAS due to infections, supportive therapy and treatment of the underlying infection is sufficient. Early recognition and appropriate management can lead to a favorable outcome in patients with cAIHA.
